# Determinants of nurse-midwives’ compliance with delayed cord clamping in primary and secondary health facilities, Tanga Region: A cross-sectional study

**DOI:** 10.1371/journal.pgph.0005603

**Published:** 2025-12-10

**Authors:** Rehema Abdallah, Fabiola Vincent Moshi, Erick Donard Oguma, Stephen M. Kibusi

**Affiliations:** 1 Department of Clinical Nursing, School of Nursing and Public Health, The University of Dodoma, Dodoma Tanzania; 2 Department of Public Health and Community Nursing, School of Nursing and Public Health, The University of Dodoma, Dodoma Tanzania; PLOS: Public Library of Science, UNITED STATES OF AMERICA

## Abstract

**Background:**

Delayed Cord Clamping (DCC) is a proven intervention that improves both maternal and newborn outcome. However, adherence to DCC remains low, and its determinants are not yet well understood.Therefore, this study aimed to assess the level of compliance with DCC and its determinants among nurse-midwives working in primary and secondary health facilities in the Tanga Region.

**Methods:**

The study employed a quantitative approach with a cross-sectional study design, conducted from 5^th^ April, 2024–15^th^ May, 2024. The multistage, clustered sampling design was employed to select both health facilities and study participants. The observation checklist and structured questionnaire, adapted from previous studies, were both used as data collection tools. A multivariable binary logistic regression model was used to assess the determinants of DDC compliance. The final results were reported using adjusted odds ratios (aORs) with their 95% confidence intervals (CI) and a significance level of p < 0.05. The data were analyzed using IBM-SPSS V. 27 software.

**Results:**

Only 30.6% of nurse-midwives were found to comply with DCC. The levels of DCC compliance among nurse-midwives were statistically significantly associated with a higher level of nurse-midwives’ education (aOR=12.26, 95% CI: 2.95-50.88), exposure to DCC-related training (aOR=5.88, 95%CI: 2.02-13.14), and availability of DCC guidelines (aOR=3.417, 95% CI: 1.331-8.768). Additionally, those who conducted 1–2 deliveries per shift (aOR=3.99, 95%CI: 1.57-10.14), and the nurse-midwives with adequate knowledge on DCC (AOR = 7.64, 95% CI: 3.04-19.17) were also found to be significantly associated with increased Odds of compliance to DCC.

**Conclusion:**

The study revealed that adequate DCC training, availability of DCC guidelines, and sufficient staffing levels per shift significantly influence nurse-midwives’ compliance with delayed cord clamping. Therefore, the government should prioritize institutionalizing regular DCC training, standardizing and disseminating updated guidelines nationwide, and investing in optimal staffing levels across primary and secondary health facilities to enhance compliance and improve maternal–newborn outcomes.

## Introduction

The timing of umbilical cord clamping is a critical component of immediate postnatal care that influences neonatal outcomes [1–4]. The World Health Organization (WHO) classifies cord clamping into two categories: Early Cord Clamping (ECC), performed within 60 seconds of birth, and Delayed Cord Clamping (DCC), performed one to three minutes after birth or when the cord pulsations cease [5]. DCC facilitates approximately 48% more blood flow from the placenta to the neonate, which is beneficial for the newborn’s health [6].

Extensive evidence highlights the maternal and neonatal health benefits of DCC. These include increased neonatal iron stores, improved hemoglobin concentration, enhanced transitional circulation, and reduced need for blood transfusions [4,7,8]. Additionally, DCC lowers the risk of necrotizing enterocolitis and intraventricular hemorrhage in preterm infants, prevents anemia, promotes neurodevelopment during childhood, and reduces postpartum hemorrhage in mothers [9,10]. Importantly, DCC is a simple, low-cost intervention that can be implemented even in health facilities with limited resources [6].

The prevalence of DCC practice varies widely between settings. Higher rates are reported in high-income countries compared to low- and middle-income countries, particularly in sub-Saharan Africa [7,8,11,12]. To maximize the benefits of DCC and reduce neonatal mortality, intensified efforts are needed in low-resource settings, supporting the achievement of Sustainable Development Goal (SDG) target 3.2, which aims to end preventable deaths of newborns and children under five years of age.

Global health authorities, including WHO, strongly recommend DCC for both term and preterm neonates, advising that cord clamping should not occur earlier than one minute after birth [5]. Despite these guidelines, barriers such as inadequate training, limited availability of DCC guidelines, resource constraints, high clinical workload, and gaps in nurse-midwives’ knowledge and attitudes toward DCC continue to impede adherence to recommended practices [12–17].

A recent systematic review and network meta-analysis evaluating four umbilical cord management strategies DCC, ECC, and umbilical cord milking demonstrated that DCC significantly reduces mortality in preterm infants compared to ECC. Moreover, DCC is associated with lower rates of intraventricular hemorrhage and reduced need for packed red cell transfusions [18**]**.

In Tanzania, despite established national guidelines advocating delayed cord clamping (DCC), data on compliance and its determinants among nurse-midwives remain limited. Previous studies have primarily focused on healthcare providers’ perceptions and experiences broadly, rather than on nurse-midwives who directly manage deliveries [19]. In Tanzania, where neonatal and infant anemia prevalence remains high, DCC may enhance neonatal iron stores and reduce the risk of anemia during early life.

While lack of DCC compliance is a central concern, this study also examines key determinants influencing adherence, including nurse-midwives’ knowledge, attitudes, training exposure, staffing levels, and clinical workload. By assessing both the level of compliance and underlying factors, this study provides evidence to inform interventions aimed at improving DCC practices in primary and secondary health facilities in the Tanga Region.

## Methods and materials

### Study setting and design

The hospital-based analytical cross-sectional study was conducted among nurse-midwives in 25 health facilities at 6 districts in Tanga region-Tanzania, from 5^th^ April 2024–15^th^ May 2024. According to the National Census 2022, the region has a population of 2,615,597 people, whereby 1,275,665 are males and 1,339,932 are females, growing at an annual average rate of 4.1% [20]. Currently, the region has 498 health facilities: 64 health centers, 412 dispensaries, and 22 hospitals [21]. Tanga region was included in this study because it is among of the regions with a high prevalence of anaemia (63.3%) among children under five 6–59 months. Also, Tanga is the leading region with high prevalence of severe anemia (5.3%) among children aged 6–59 months [22].

### Study population

The study population were the nurse-midwives who working at labor ward in primary and secondary health facilities in Tanga Region.

### Inclusion criteria

Registered or enrolled nurse-midwives working in the labour ward were included. A minimum of 6 months’ maternity ward experience was considered, to ensure adequate exposure to maternity guidelines, recommendations, and relevant training.

### Exclusion criteria

Nurse-midwives who declined to participate or withdrew consent, and those who were on leave or otherwise unavailable during the data collection period were excluded.

### Sample size determination

Because the target population of eligible nurse-midwives was finite and known (N = 243), we used the Yamane formula for finite populations to obtain a feasible base sample at 95% confidence and 5% precision: no = N/(1 + Ne^2^)=243(1 + 243(0.05)2)=2431.6075 ≈ 151. Because the study employed a multistage (clustered) sampling design, where nurse-midwives are clustered within health facilities and thus may exhibit correlated behaviors, we applied a design effect (DEFF) of 1.30 to inflate the sample size to account for the increased variance that clustering introduces, a commonly used conservative value for cluster sampling in similar health services studies. The final required sample was therefore n = no x DEFF = 151 × 1.30 = 196.3, rounded to 196. This approach (Yamane for finite population plus a design effect) appropriately accounts for both the small target population and the anticipated intra-cluster correlation.

### Sampling procedure

The study employed a multistage, clustered sampling design to select both health facilities and study participants. In the first stage, Tanga Region was purposively selected. A census was conducted to list all 11 districts within the region, from which six districts were randomly selected using the lottery method. All health facilities within these six districts were then listed and stratified into two categories based on their level of care: primary (health centers and district hospitals) and secondary (regional referral hospital). From the secondary stratum, the only regional referral hospital in the region was purposively included. From the primary stratum, six district hospitals and 18 health centers were selected by simple random sampling using the lottery method with replacement.

In the second stage, a proportionate allocation method was applied to determine the number of nurse-midwives to be recruited from each selected facility based on the size of its labor ward staff. Within each facility, participants were then randomly selected using a simple random sampling technique until the minimum required sample size of 196 was achieved.

### Data collection method

An observational checklist and self-administered questionnaire were used to gather data from study participants. The principal investigator and research assistants were used to collect data from study participants. The research assistants received two days orientation training prior to data collection on data collection techniques and research regulations, including respect for participant autonomy, privacy, confidentiality. After training the evaluation was done to see if the research assistant have mastered the questionnaire and interview techniques. The lead investigator’s responsibilities included providing all study materials, supervising and training research assistants, and overseeing the entire data gathering procedure.

### Data collection tools

The observational checklist and structure questionnaire adapted from previous studies were used as a data collection tools [5,14]**.** The observational checklist was used to assess the level of compliance on delayed cord clamping and it composes of 5 items with binary response. The structured questionnaire was used to assess the nurse-midwives’ knowledge on DCC, attitude, and self-reported institutional factors. The institutional factors were added on the adapted questionnaire, to account factors associated with DCC. The questionnaire comprised four sections, the first section comprises 6 items with multiple responses to assess demographic and professional characteristics, the second section comprised 5 items with binary response to assess nurse-midwives’ level of knowledge on DCC, the third section comprises 5 items with 5-point Likert scale to assess level of attitude toward DCC, and the fourth section comprised 7 items with binary response to assess self-reported institutional factors.

### Validity and reliability

**Instrument adaptation:**

The observational checklist and questionnaire were adapted from validated tools used in similar DCC studies [5,14]. Adaptation involved:

a) Rewording items to fit Tanzanian maternal health terminology.
b) Adding new questions on institutional factors specific to the Tanga context (supply availability, staffing patterns).
c) Aligning clinical practice definitions with WHO guidelines for delayed cord clamping.

**Expert review (content validity):**

Two senior maternal health experts (one obstetrician, one midwifery educator) reviewed the draft tools. They assessed:

a) Relevance (whether items measured intended constructs).
b) Clarity (language, cultural appropriateness).
c) Completeness (whether any key aspects were missing).

Suggestions included simplifying medical jargon, rearranging questions for logical flow, and refining binary responses for observational items. And revisions were made before pretesting.

**Pretesting (face validity):**

a) Conducted in non-study facilities with 10% of the final sample (n ≈ 20).
b) Aim: to test comprehension, time taken to complete, and response consistency.
c) Participants completed the questionnaire, followed by short debrief interviews to identify confusing wording or ambiguous response options.
d) Observations of DCC were piloted to check the feasibility and consistency in timing measurements.
  **Evaluation and final adjustments:** Feedback from pretest revealed two knowledge questions that were prone to misinterpretation; these were rephrased. Some Likert-scale statements were reversed to reduce acquiescence bias. The estimated completion time reduced from ~25 to ~18 minutes after removing redundant items.
  **Reliability testing:** Internal consistency assessed using Cronbach’s alpha: Institutional factors = 0.886, Attitude = 0.700, and Knowledge = 0.725. All met the Cronbach’s alpha ≥ 0.7 threshold for acceptable reliability.

### Variables description and measurement

Refer Table 1

**Table 1:** Variables description and measurement.

Variable	Definition & Measurement	Cut-off/ Categorization
**Dependent Variable**: Compliance with DCC	Observed timing of cord clamping	WHO criteria: Complied if clamped after 1–3 min or when pulsations stop; Not complied if clamped <60 sec
**Knowledge**	5 binary items (Yes = 1, No = 0), total score range 0–5	Score ≥ 3 = Adequate knowledge [13].
**Attitude**	5 items on a 5-point Likert scale (1 = Strongly Disagree to 5 = Strongly Agree), total score 5–25	Score above the mean = Positive attitude [13].
**Institutional Factors**	7 binary items (Yes = 1, No = 0) assessing facility resources, policies, and staffing	≥1 correct response = Factor present
**Demographic & Professional Characteristics**	Age group, sex, education level, maternity experience, professional registration, facility ownership	Categorical variables

### Data analysis

Data were cleaned, managed, and analyzed using IBM SPSS Statistics Version 27. Descriptive statistics including means, frequencies, and standard deviations were used to summarize participants’ social demographic and professional characteristics, knowledge of DCC, attitudes toward DCC, and self-reported institutional factors. Multivariable binary logistic regression analysis was performed to assess the strength of associations between nurse-midwives’ compliance with DCC and potential predictor variables. Results from the regression analysis are presented as adjusted odds ratios (aOR) with 95% confidence intervals (CI), considering p-values < 0.05 as statistically significant.

### Ethical consideration

The ethical approval to conduct this study was granted by the University of Dodoma’s Institutional Research Review Committee Board by the letter issued on 9^th^ February 2024; with Ref. No.MA.84/261/02/’A’/69/98. Both verbal and written consent were sought from the respective participants who were involved in this study. The participants’ values, dignity, and integrity was safeguarded. All participants were ensured anonymity and confidentiality, and no participants were forced to participate in this study.

## Results

### Social demographic and professional characteristics of study participants (n=196)

The study included a minimum of 196 nurses-midwives from primary and secondary health facilities in Tanga Region, with a 100% response rate. The mean age of study participants was (37 ± 10.2) years, with the minimum and maximum age being 18 and 56 years, respectively. From Table 2, approximately three-quarters of the nurse-midwives 137(69.9%) were female. Majority of nurse-midwives 184(93.9%) have diploma and certificates, and were fairly distributed. Moreover, about 113(57.7%) of nurse-midwives were registered nurses (RN). Additionally, nearly three-quarter of nurse-midwives 146(74.5%) have maternity experience ranging (1–10) years, and about two-third 122(62.2%) were working at public health facilities. Mostly of the nurse-midwives were working at district hospitals and health centers, which were accounted 86(43.9%) and 90(45.9%), respectively.

**Table 2:** Social demographic and professional characteristics of study participants (n = 196).

Variables	Frequency (n)	Percent (%)
**Age groups**		
<25 years	8	4.0
26-35 years	84	42.9
36-45 years	59	30.1
46-55 years	35	17.9
>56 years	10	5.1
**Sex**		
Male	59	30.1
Female	137	69.9
**Education level**		
Certificate	88	44.9
Diploma	96	49.0
Bachelor	10	5.1
Masters	2	1.0
**Maternity experience**		
6-11 months	23	11.7
1-5 years	76	38.8
6-10 years	70	35.7
>10 years	27	13.8
**Professional nurse registration**		
Enrolled nurse	83	42.3
Registered nurse	113	57.7
**Participants per health facility**		
Region hospital	20	10.2
District hospital	86	43.9
Health center	90	45.9
**Participants per organization ownership**		
Public hospital	122	62.2
Private hospital	74	37.8

### Level of compliance on delayed cord clamping among nurse-midwives (n=196)

Of the 196 nurse-midwives surveyed, 30.6% complied with DCC, while 69.4% did not comply on DCC. The majority of study participants (66.9%) clamping cord immediately after delivery, and only 21.3% clamped the cord early for resuscitation, of whom 6.6% required implicit consent to use DCC, with just 5.2% clamping the cord before cessation of pulsation.

### Self-reported institutional factors on delayed cord clamping among nurses-midwives (n=196)

The findings revealed that, about 62(31.7%) of the Nurse-midwives reported exposure to training, availability of guidelines or DCC protocols 41(20.9%), low delivery volume per shift 35(17.9%), adequate number of staff per shift 20(10.2%), adequate medical supplies and equipment’s 17(8.2%), and minimal number of emergency deliveries 10(5.1%) may influence their compliance on Delayed Cord Clamping.

### Level of knowledge on delayed cord clamping among nurse-midwives (n=196)

The finding revealed that more than half of nurse-midwives 196(52.0%) had inadequate knowledge on Delayed Cord Clamping. The majority of study participants 129(65.8%) were unable to recognize the benefits of Delayed Cord Clamping. Also, approximately 166(84.7%) were unaware if delayed cord clamping is a reasonable option for term and pre-term delivery. Furthermore, the majority 168(85.8%) were not informed that delayed cord clamping does not increase the risk of postpartum hemorrhage or need of blood transfusion. Also, about 155(79.1%) declared that Early Cord Clamping is only for resuscitation and prevent postpartum hemorrhage, as illustrated in Table 3.

**Table 3:** The nurse-midwives responses regarding knowledge on delayed cord clamping (n = 196).

Statements	Frequency (n)	Percent (%)
Delayed cord clamping is reasonable option for term and pre-term deliveries.		
Yes	30	15.3
No	166	84.7
Early cord clamping is only for resuscitations and prevent post-partum hemorrhage		
Yes	155	79.1
No	41	21
Delayed cord clamping has benefit for pre-term and term neonates of give iron store		
Yes	129	65.8
No	67	34.2
Delayed cord clamping is a viable choice for all deliveries		
Yes	30	15.3
No	166	84.7
Delayed cord clamping does not increase the risk of post-partum hemorrhage or need of blood transfusion		
Yes	28	14.2
No	168	85.8

### Level of attitude on delayed cord clamping among nurse-midwives (n=196)

The study revealed majority of study participants 125(63.8%) had negative attitude toward Delayed Cord Clamping. Table 4 shows that, about half of the participants 101(51.1%) were not sure if a Delayed Cord Clamping can help to increase iron store for newborn. Also, about 105(53.6%) of participants were not sure if a Delayed Cord Clamping can stabilize transitional blood circulation. Moreover, about 119(59.9%) were disagreed that Delayed Cord Clamping has benefit to neonates with severe birth asphyxia.

**Table 4:** Attitude of nurse-midwives toward Delayed Cord Clamping (n = 196).

Items	SD	D	NS	A	SA
	n (%)	n (%)	n (%)	n (%)	n (%)
1. DCC is good for preterm neonate who does not require positive pressure ventilation.	11(5.6)	43(21.9)	87(44.4)	37(18.9)	18(9.2)
2. DCC help to increase iron store for neonates.	6(3.1)	26(13.3)	101(51.1)	37(18.9)	26(13.3)
3. DCC can stabilize transitional blood circulation.	7(3.6)	20(10.2)	105(53.6)	53(17.9)	29(14.8)
4. DCC prevent anemia in neonates and childhood	4(2.0)	20(10.2)	99(50.5)	39(19.9)	34(17.3)
5. DCC is beneficial to neonates with severe asphyxia.	78(39)	41(20.9)	34(17.3)	26(13.3)	17(8.7)

**SD = Strongly disagree, D = Disagree, N = Neutral, Agree = 4, SA = Strongly agree.**

### Determinants of compliance with delayed cord clamping among nurse-midwives in primary and secondary health facilities, Tanga Region (n = 196)

Bivariable and multivariable binary logistic regressions analysis were used to establish the strength of association between social demographic and professional characteristics, reported institutional factors, knowledge, and attitude against the DCC compliance. After adjusting confounders, in multivariable binary logistic regression analysis, the finding revealed that Nurse-midwives with higher level of education (aOR= 12.26, 95% CI: 2.95-50.88; p < 0.001) were 12 times more likely to comply on DCC than those with diploma level. Similarly, nurse midwives with diploma level (aOR= 4.45, 95% CI: 1.68-11.79; p = 0.003) were 4 times more likely to comply with DCC than those with certificate level. Also, Nurse-midwives with exposure to DCC training (aOR= 5.88, 95%CI: 2.02-13.14; p = 0.001) were 6 times more likely to comply with DCC than those with no exposure to DCC training.

Furthermore, Nurse-midwives who were reported the availability of DCC guideline or protocols (aOR= 3.42, 95% CI: 1.33-8.77; p < 0.001) were 3 times more likely to comply on DCC than those who did not report. Similarly, Nurse-midwives who conducted 1–2 delivery per shift were 4 times more likely to comply on DCC than those who conducted more than 2 deliveries (aOR= 3.99, 95%CI: 1.57-10.14, p = 0.004). Lastly, Nurse-midwives with adequate knowledge on DCC (aOR= 7.64, 95% CI: 3.04-19.17; *p* < 0.001) were 7 times more likely to comply with DCC than their counterpart. Refer Table 5.

**Table 5:** Bivariable and Multivariable Binary Logistic Regression Analysis of Determinants of Nurse-Midwives’ Compliance with Delayed Cord Clamping (n = 196).

Variables	cOR	95% CI		p-value	aOR	95% CI		p-value
		Lower	Upper			Lower	Upper	
**Professional Education**								
Certificate	Ref				Ref			
Diploma	2.575	1.272	5.212	0.009	4.449	1.678	11.792	**0.003**
Higher level	7.583	2.674	21.507	<0.001	12.257	2.953	50.879	**0.001**
**Maternity experience**								
<1 year	Ref				Ref			
>1–5 years	2.589	0.919	7.294	0.072	1.091	0.281	4.237	0.900
>5 years	7.00	2.393	20.480	<0.001	2.733	0.648	11.526	0.171
**Number of deliveries per shift**								
1-2 deliveries	2.188	1.171	4.086	0.014	3.991	1.572	10.135	**0.004**
More than 3 deliveries	Ref				Ref			
**Exposure to DCC training**								
No	Ref				Ref			
Yes	7.026	3.565	13.849	<0.001	5.150	2.018	13.142	**0.001**
**Available site for documentation**								
No	Ref				Ref			
Yes	4.143	2.059	8.334	<0.001	2.507	0.919	6.843	0.073
**DCC guideline and protocols**								
No	Ref				Ref			
Yes	3.517	1.684	7.344	0.001	3.417	1.331	8.768	**0.011**
**Knowledge on DCC**								
Inadequate knowledge	Ref				Ref			
Adequate knowledge	3.769	1.965	7.231	<0.001	7.637	3.044	19.165	**<0.001**

## Discussion

This study found that a substantial proportion of nurse-midwives in primary and secondary health facilities in the Tanga Region do not fully comply with delayed cord clamping (DCC) guidelines. Observations revealed that most nurse-midwives practiced immediate cord clamping, with only a small fraction performing DCC according to recommended timing. Compliance was influenced by several factors, including higher educational level, exposure to DCC-related training, availability of DCC guidelines, adequate knowledge of DCC, and conducting fewer deliveries per shift. These findings highlight persistent gaps between guideline recommendations and actual practice, emphasizing the need to address both individual and institutional determinants to improve neonatal outcomes.

The majority of nurse-midwives were female, which aligns with previous studies conducted in Zambia and Saudi Arabia, both showing that the nursing field is predominantly female, with approximately three-quarters being women [23,24]. This sex distribution reflects the global trend and is consistent with findings from Kenya by Kabo et al. (2022), who also reported female predominance in nursing and midwifery professions [25].

Additionally, most nurse-midwives were aged between 26 and 45 years and held diplomas. These findings are consistent with studies by Kabo et al. (2022) and Ibrahim et al. (2017), which reported similar age groups and education levels among nurse-midwives [23,24]. Similarly, Abd et al. (2024) found that over one-third of nurses were aged 30–34 years [26]. Our study also found that most nurse-midwives working in public health facilities were registered professionals with one to ten years of maternity experience, corroborating findings by Ramadhan et al. (2020) [16].

Significant associations were observed between several social demographic and institutional factors and compliance with DCC. Educational level was strongly linked to compliance, consistent with prior studies showing that nurse-midwives with higher education (bachelor’s or master’s degrees) were more likely to comply with DCC protocols [13,27]. However, this contrasts with Ramadhan et al. (2020), who reported no significant association [16]. These differences may be due to variations in health system capacity, availability of resources, and access to ongoing professional development across settings.

Compliance was also significantly associated with DCC-related training, availability of guidelines, adequate knowledge, and lower delivery workload per shift. Similar findings were reported by Boerre et al. (2015) [28]. In resource-constrained settings like Tanzania, shortages of staff and inconsistent guideline dissemination may hinder the implementation of WHO recommendations compared to more resourced settings such as Saudi Arabia.

Regarding knowledge, most participants demonstrated inadequate understanding of DCC. This may be attributed to negative attitudes toward DCC practice, lack of accessible guidelines, and limited routine training. These findings contrast with studies reporting that most nurses understood the benefits of DCC [13–15]. The variation likely reflects differences in guideline availability and training frequency.

Concerning attitude, many nurse-midwives exhibited negative attitudes toward DCC. This aligns with previous studies reporting that over half of nurse-midwives held negative attitudes toward DCC [17,19,29]. These results suggest limited acceptance of DCC and its benefits among Tanzanian nurse-midwives. In contrast, a study in Saudi Arabia found that nearly three-quarters of nurse-midwives believed in the benefits of DCC for term and preterm infants [30]. Differences in resource availability, training, and institutional support may explain these contrasting findings.

Efforts to improve routine DCC training and ensure widespread availability of guidelines could enhance awareness, positively influence attitudes, and improve DCC practice in primary and secondary health facilities.

Regarding institutional factors, fewer than half of participants reported the availability of DCC guidelines, lower delivery volumes per shift, and exposure to training, all of which may influence compliance. This indicates that institutional support for DCC remains low. The findings also suggest that adequate staffing and guideline availability are critical to improving DCC compliance, corroborating studies by Mwamba (2022) and Baker et al. (2015) [24,29]. Effective guideline distribution within departments appears essential for promoting compliance [7,31].

High delivery volume per shift negatively affected DCC implementation, consistent with previous research indicating that workload and multitasking reduce adherence to guidelines [16]. These results highlight the need to ensure adequate staffing levels. Training and education on DCC are vital to maintaining safe and effective cord clamping practices [32,33].

### Theoretical interpretation

The study’s findings characterized by inadequate knowledge, negative attitudes, and low levels of compliance with delayed cord clamping (DCC). This can be critically interpreted through the lens of the Theory of Planned Behavior (TPB). According to TPB, behavior is influenced by three main constructs: attitude toward the behavior, subjective norms, and perceived behavioral control [34]. The negative attitudes observed among many nurse-midwives suggest that DCC is not yet fully accepted or valued within the prevailing professional culture. Combined with low knowledge levels, this undermines the formation of strong behavioral intentions to perform DCC.

Moreover, even in cases where nurse-midwives may express some intention to comply with guidelines, perceived behavioral control which includes perceived ability to perform the behavior given external constraints emerged as a significant limiting factor. Participants cited high workloads, inadequate staffing, and lack of clear institutional support as barriers, all of which reduce the likelihood that intentions translate into actual practice. These findings suggest that both motivational (attitude-related) and capability-related (control-related) dimensions are weak, leading to low DCC compliance.

To further unpack the structural drivers of poor implementation, the WHO Health Systems Building Blocks framework provides a useful macro-level perspective [35]. Weaknesses were evident across several key system components: health workforce, due to staffing shortages and insufficient training; service delivery, marked by inconsistent practice standards; and access to medical products and guidelines, with many facilities lacking current or visible protocols on DCC. The misalignment between individual-level readiness and system-level support structures creates an environment where evidence-based practices like DCC are unlikely to be routinely implemented.

Together, these frameworks highlight the complexity of behavior change in clinical settings. Addressing knowledge gaps and reshaping attitudes are necessary but not sufficient conditions for change. Structural interventions such as improving health system capacity, strengthening guideline dissemination, and creating an enabling environment are equally crucial. The findings support a holistic approach to implementation that bridges behavioral theories and health systems thinking.

## Policy implications

The findings underscore the need for policy interventions that go beyond traditional training models and address both the behavioral and structural determinants of clinical practice. To counteract negative attitudes and low knowledge levels among nurse-midwives, policymakers should prioritize the integration of delayed cord clamping (DCC) into pre-service education and strengthen in-service training programs with a focus not only on technical knowledge but also on behavior change strategies. Training should be designed to shift attitudes, reinforce the clinical value of DCC, and cultivate professional norms that support its routine practice. Additionally, fostering positive subjective norms through mentorship, peer learning, and leadership engagement can help shift workplace culture toward greater acceptance of DCC as standard care.

At the systems level, action is needed to enhance perceived and actual behavioral control among healthcare providers. This includes addressing workforce shortages, improving task allocation, and reducing workload pressures that currently inhibit practice change. Ensuring the routine availability and visibility of updated DCC guidelines in all maternity care settings is essential to support decision-making at the point of care. Furthermore, embedding DCC indicators into maternal and newborn health quality improvement frameworks such as supervision tools, audits, and feedback loops can institutionalize accountability and encourage sustained adherence. These interventions must be implemented as part of a coordinated, system-wide strategy that acknowledges the interaction between individual readiness and institutional capacity. Only by addressing both domains can policies effectively improve DCC uptake and, ultimately, neonatal outcomes.

## Limitations of the study

This study has several limitations that should be acknowledged:

This study has several limitations that should be considered when interpreting the findings. First, the use of self-reported data to assess nurse-midwives’ knowledge, attitudes, and perceptions of institutional factors introduces the potential for recall bias and social desirability bias. Participants may have over reported positive behaviors or knowledge to align with perceived expectations, thereby affecting the accuracy of the data.

Second, the observational data on compliance with delayed cord clamping practices may have been influenced by the Hawthorne effect. Since participants were aware that they were being observed, their behavior may have changed temporarily, leading to an overestimation of compliance levels during the study period.

Third, the study sample was drawn from selected districts within a single region of Tanzania. This geographic limitation restricts the generalizability of the findings to other regions of the country. Differences in health system infrastructure, facility types, and the diversity of the healthcare workforce across regions may influence both practices and perceptions related to Delayed Cord Clamping.

Finally, the classification of nurse-midwives’ knowledge and attitudes was based on mean scores or arbitrary cut-off points, rather than on validated instruments or thresholds established through pilot testing. This limits the precision and reliability of the classifications. Future research should consider the development and use of locally validated assessment tools to ensure more accurate and context-appropriate evaluations of knowledge and attitudes.

## Future research

Larger, nationally representative studies are recommended to enhance external validity and provide a more comprehensive understanding of DCC compliance across diverse settings in Tanzania. Implementation research applying behavioral and health systems frameworks could identify targeted interventions to address both individual-level and institutional barriers to DCC.

## Supporting information

S1 Data Anonymized raw dataset on nurse-midwives’ compliance with delayed cord clamping. (SAV)
